# Cystic renal cell carcinoma: a report of 67 cases including 4 cases with concurrent renal cell carcinoma

**DOI:** 10.1186/1471-2490-14-87

**Published:** 2014-11-07

**Authors:** Shanwen Chen, Baiye Jin, Liqi Xu, Guanghou Fu, Hongzhou Meng, Ben Liu, Jun Li, Dan Xia

**Affiliations:** Departments of Urology, The First Affiliated Hospital, Medical School of Zhejiang University, Hangzhou, Zhejiang 310031 P.R. China; Departments of Pathology, The First Affiliated Hospital, Medical School of Zhejiang University, Hangzhou, Zhejiang 310031 P.R. China; Department of Urology, The First Affiliated Hospital of Medical College, Zhejiang University, No. 79 Qing Chun Road, HangZhou, 310003 China

**Keywords:** Cystic renal cell carcinoma, Diagnosis, Nephrectomy, Partial nephrectomy

## Abstract

**Background:**

Cystic renal cell carcinoma (CRCC) is relatively rare; CRCC is frequently misdiagnosed as a benign renal cyst. CRCC carries an excellent prognosis following surgical treatment. The aim of our study was to summarize the management of CRCC and to characterize the prognosis of affected patients.

**Methods:**

A retrospective study of 67 patients with CRCC was conducted at our center between January 2005 and April 2013. Patient prognosis as well as the clinical manifestations, imaging characteristics, treatment, and pathologic features of CRCC were summarized based on available medical record data.

**Results:**

We identified 67 cases of CRCC, representing 2.5% of all renal cell carcinoma cases. The tumor was discovered incidentally in 70% of the cases. Ultrasonography was found to be a useful screening tool, but computed tomography remains the imaging study of choice for identifying malignant features. Magnetic resonance imaging can be used in equivocal cases. Regarding treatment, radical nephrectomy was performed in 52% of the cases, and partial nephrectomy was selected in the remaining 48% of cases. None of the 46 patients (68% of the study group) available for follow-up showed any evidence of recurrence.

**Conclusions:**

CRCC is an uncommon subtype of renal cell carcinoma, occurring in 2.5% of cases. CRCC carries an excellent prognosis after surgical treatment. Partial nephrectomy should be regarded as the preferred surgical technique for CRCC.

## Background

Cystic renal cell carcinoma (CRCC) is relatively rare; it represents a special subtype of renal cell carcinoma (RCC) associated with a low nuclear grade and stage and an excellent prognosis regardless of tumor size. However, CRCC is frequently misdiagnosed as a benign renal cyst because it shares similar clinical manifestations and imaging characteristics. In the present study, we retrospectively analyzed 67 CRCC cases treated at our center; we summarize patient prognosis and the clinical manifestations, imaging characteristics, treatment, and pathologic features of CRCC based on medical record data. To our knowledge, this represents the largest series of CRCC cases, and it includes four cases with other concurrent RCCs.

## Methods

We reviewed our center’s pathology files and identified all RCC cases with a cystic component occurring between January 2005 and April 2013; 67 cases of CRCC were identified. Clinical data, including clinical and radiographic characteristics, surgical management, pathologic features, and outcomes, were retrospectively reviewed.

Prior to surgery, patients underwent renal ultrasonography, abdominal computed tomography (CT), and magnetic resonance imaging (MRI) for evaluation of each patient’s overall and renal status. All cystic masses were graded according to the Bosniak classification system [1], and the pathological specimens were staged according to the tumor-node-metastasis (TNM) classification for RCC [2]. Additionally, a tumor nuclear grade was assigned using the Fuhrman system [3]. All histopathologic slides were reanalyzed by a single pathologist specialized in genitourinary pathology. The size of the neoplasm was measured grossly using the maximum diameter. Clinical follow-up data was obtained from patients’ records as well as referring physicians; telephone interviews were conducted for patients who lacked clinical follow-up within the past 6 months. Finally, all data were analyzed using either the Student’s t-test or a chi-square test.

The Medical Ethics Committee of the First Affiliated Hospital of Medical College, Zhejiang University approved this retrospective study; the study was exempted from the requirement to obtain informed consent from the other 63 patients (not including 4 cases of concurrent RCCs) in consideration of the nature of the study. Written informed consent was obtained from the 4 patients (4 cases of concurrent RCCs) for publication of this manuscript and accompanying images.

## Results

**Figure 1:**
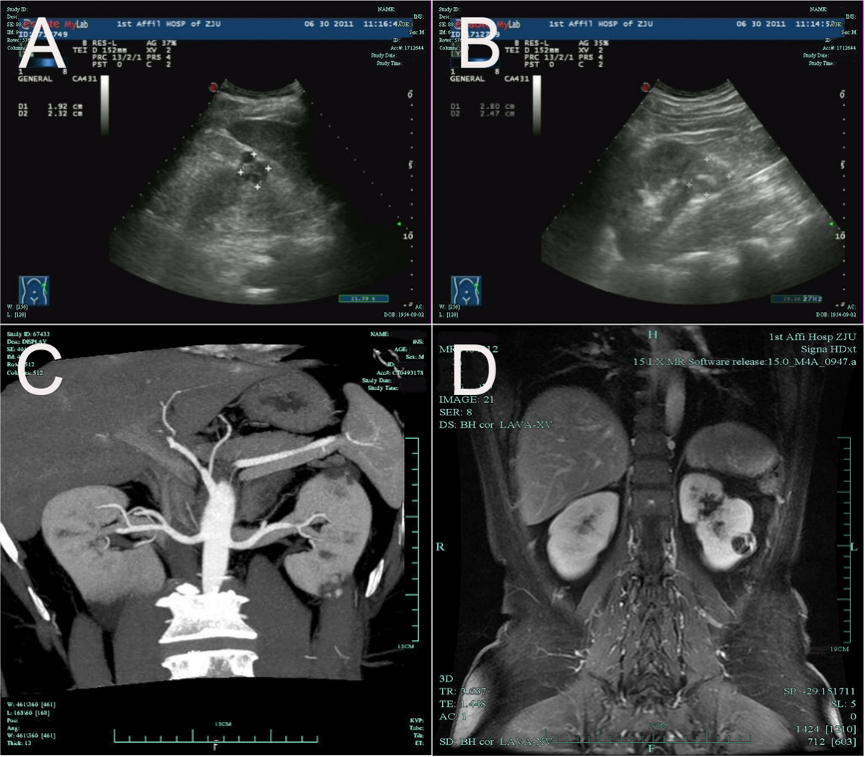
**Ultrasound, computed tomography, and magnetic resonance images. A)** Ultrasound: Upper pole complex cystic renal mass (patient 2). **B)** Ultrasound: Lower pole renal solid mass (patient 2). **C)** Computed tomography: Lower pole renal mass with a concurrent cyst in the upper pole (patient 2). **D)** T1-weighted magnetic resonance imaging: Complex cystic mass with thick and irregular enhancing cyst walls in the middle part of the kidney.

We identified 67 cases of CRCC, which represented 2.5% of the 2679 cases of RCC resected at our institution between January 2005 and April 2013; 217 out of all 2679 RCC patients had concurrent cystic renal lesions, and 4 out of the 67 CRCC patients had other concurrent RCCs (Tables 1 and 2). For the 67 patients diagnosed with CRCC on final pathology, the average age at diagnosis was 56.0 years, with a range of 24–83 years; the average age was 58.2 years (n =46) for men and 54.7 years for women. The tumor was incidentally found during evaluation for an unrelated condition in 47 patients (70%), whereas a renal tumor was suspected in 20 (30%). Left renal cystic masses were identified in 32 patients; the remaining 35 patients had right renal cystic masses. The masses were located in the upper pole in 24 patients, the lower pole in 28, and other locations in the remaining 15. Renal ultrasound scans were available in all 67 cases (Figure 1A, B) and demonstrated a complex cystic mass in 49 cases. Renal CT scans were performed in 62 patients (Figure 1C), and a possible CRCC was reported in 48. Additional investigation included MRI in 21 patients (Figure 1D); an enhancing, cystic renal lesion suggestive of malignancy was identified in 18.

**Figure 2:**
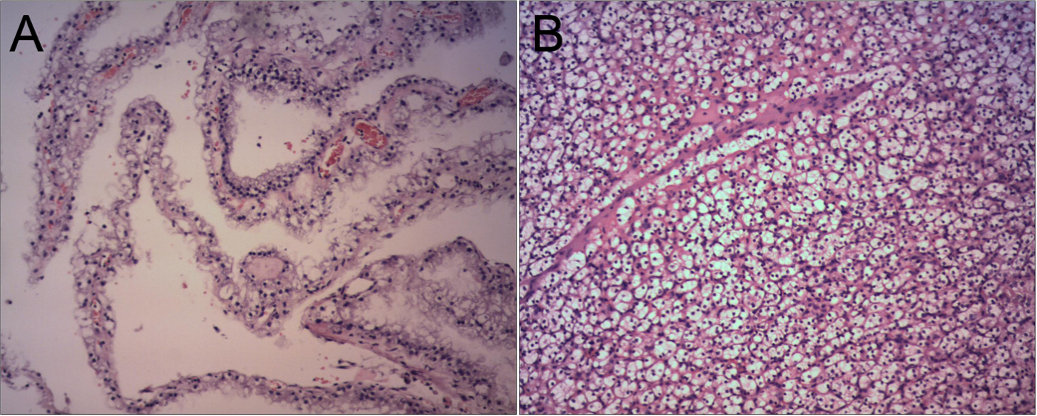
**Pathological images from patient 2. A)** Cystic renal cell carcinoma. Cysts are lined by clear cells with uniform nuclei. Clusters of clear cells are present in the septal walls. **B)** Renal clear cell carcinoma. The tumor consists of sheet-like, solid clear cells. H&E × 100.

The patients underwent the following surgical procedures: open radical nephrectomy (n =19), open partial nephrectomy (n =12), laparoscopic radical nephrectomy (n =9), laparoscopic partial nephrectomy (n =20), and a second anesthesia for radical nephrectomy (n =7, including 3 patients with an intraoperative frozen section diagnosis of a renal cyst and 4 patients without intraoperative frozen section analysis; the final pathological result was CRCC for all of these cases). Prior to 2010, 3 of 14 patients underwent partial nephrectomy. After 2010, 29 of 53 cases underwent partial nephrectomy. Fifty-three cases (79%) were nuclear grade I, 12 cases (18%) were nuclear grade I-II, and 2 (3%) were nuclear grade II (Figure 2). Among the 67 patients, 25 (37%) had stage T1a disease, 35 (52%) had stage T1b disease, and 7 (10%) had stage T2a disease.

**Table 1:** **The character of cystic renal cell carcinoma**

Features	Value	%
Age at the time of diagnosis (yr)		
Median	56	
Range	24-83	
Sex		
Female	21	31
Male	46	69
Side		
Left	32	48
Right	35	52
Pole		
Upper pole	24	36
Lower pole	28	42
The remaining	15	22
Surgery		
Open radical nephrectomy	19	28
Open partial nephrectomy	12	18
Laparoscopic radical nephrectomy	9	13
Laparoscopic partial nephrectomy	20	30
Two operations	7	10
Fuhrman nuclear grade		
I	52	78
I-II	12	18
II	3	5
TNM stagea		
T1a	25	37
T1b	35	52
T2a	7	10

**Table 2:** **The character of cystic renal cell carcinoma concurrent renal cell carcinomas**

Pt. No.	Age (yr)	Sex	Mass location (size)	Side/Treatment	Follow-up (mo)	Outcome
1	48	M	Upper pole: cystic(4.5 × 3.0 cm)	Left radical nephrectomy	36	No evidence of disease
			Lower pole: solid(2.8 × 2.0 cm)			
2	59	M	Upper pole: cystic(2.5 × 2.0 cm)	Left partial nephrectomy	29	No evidence of disease
			Lower pole: solid(3.5 × 3.0 cm)			
3	50	F	Mid pole: solid (2.0 × 2.0 cm)	Right radical nephrectomy	8	No evidence of disease
			Lower pole: cystic (4.0 × 4.0 cm)			
4	37	M	Mid pole: solid(3.0 × 3.0 cm)	Left partial nephrectomy	8	No evidence of disease
			Mid pole: cystic(2.0 × 2.0 cm)

All patients had uneventful surgical recoveries. Forty-six patients were followed for a mean time of 42 months (range: 6–84 months); among these, 7 patients (who were initially misdiagnosed as having benign tumors on preoperative or intraoperative pathology but confirmed as having malignant tumors on postoperative pathology) were followed for 56 months (range: 35–77 months). Their overall outcomes were excellent: there was no evidence of neoplastic recurrence or metastasis. Four cases died of non-neoplastic diseases.

## Discussion

Cystic degeneration of the kidney is very common among renal lesions. However, CRCC is rare, accounting for only 1% to 4% of all RCCs in previous studies [4–6]; in our series, CRCC represented 2.5% of the RCCs. Corica et al. found that 83% of CRCC cases were diagnosed incidentally [5]. The results of our study are in agreement, showing that CRCC is more likely to be discovered incidentally (on abdominal imaging for unrelated conditions) than as a result of tumor-related symptoms.

Accurate diagnosis is sometimes difficult because CRCC, conventional RCC with cystic change, and benign renal cystic disease have similar imaging characteristics. Ultrasonography has been found to be a useful screening tool. On sonography, CRCC appears as a cystic or cystic-solid structure with thick capsule walls, hyperechoic internal septa, and heterogeneous echogenicity [7, 8]; duplex Doppler ultrasound may or may not display increased blood flow in the walls of the cyst. Computed tomography can provide richer diagnostic information than ultrasonography. On CT, CRCC appears as a cystic or mixed cystic-solid mass with thick and irregular enhancing cyst walls, with or without calcification. According to the literature, thick calcification or crescent calcification has more significance in the diagnosis of CRCC. The septa tend to be of uneven thickness (they are often >1 mm in diameter), and nodular thickening can appear at the junctions with the capsule walls. The cystic fluid contains debris, flocculent particles, and blood clots, giving it an uneven appearance on CT scan. The lesions typically have unclear borders adjacent to the renal parenchyma. In our study, the lesions were described as suggestive of CRCC in 48 out of the 67 patients. Computed tomography is less reliable in evaluating CRCC lesions under 4 cm in size, because a small lesion may appear to be a solid mass [9]. Furthermore, CT may not be able to differentiate CRCC from necrotic RCC [10]. If the determination of benign versus malignant renal cystic disease is unclear, MRI evaluation can provide additional information. We found MRI to be superior to CT for such differentiation, as did Adey el al. in a previous study [11].

The differential diagnoses for CRCC include RCC with cystic change, hereditary leiomyomatosis manifesting as a cystic renal lesion, cystic nephroma, clear cell papillary RCC, and other cystic lesions of the kidney. These cystic tumors are extremely difficult to distinguish based on clinical, radiological, and gross features, and they can cause a diagnostic dilemma. Cystic renal cell carcinoma is a predominantly cystic lesion with a small solid component (25% or less). Renal cell carcinoma usually presents as a solid mass; however, in 10–22% of cases, it appears as a unilocular or multilocular cystic mass on imaging studies. Four mechanisms have been described to account for RCC with cystic features: intrinsic unilocular cystic growth (papillary cystic adenocarcinoma), intrinsic multilocular cystic growth, tumor necrosis resulting in cyst formation (pseudocyst), and tumor origination in a preexisting simple renal cyst. Typically, hereditary leiomyomatosis-associated renal tumors display type 2 papillary architecture, but they can show a variety of patterns, including cystic, tubular-papillary, tubular, and solid. Cystic nephroma consists of a circumscribed mass of cysts with intervening fibrous septa, occasionally areas of calcification, and regions of cellular bland ovarian-like stroma. The cysts are lined by a single layer of flattened low cuboidal or hobnail benign epithelium. Clear cell papillary RCC is usually cystic, with cyst walls lined by clear cells; however, much of the tumor typically exhibits papillary architecture, a feature not found in CRCC. It is important to avoid misdiagnosing CRCC as conventional clear cell RCC, which is one of the reasons that we chose to present this series of CRCC cases.

Because of potential tumor rupture or spillage, the traditional treatment for CRCC has been radical nephrectomy. However, CRCCs tend to be smaller tumors at initial diagnosis and to have a lower T stage and nuclear grade; therefore, these lesions may be more amenable to partial nephrectomy. Because of the benign nature of CRCC, along with accumulating experience with partial nephrectomies and improved surgical techniques, increasing numbers of surgeons are choosing partial nephrectomy as the first-choice therapy for CRCC. Gong et al. suggested that a partial nephrectomy should be considered when a complex multicystic renal mass with enhanced density is observed, particularly as CRCC — like conventional RCC — is often located in the renal polar regions, which makes a partial nephrectomy approach feasible [12]. Based on their recent findings, You et al. indicated that 96% of patients with benign cysts or CRCCs greater than 4 cm in size might be able to avoid radical nephrectomy and instead undergo partial nephrectomy [13]. At our center, partial nephrectomy has been widely performed for renal carcinoma since 2010. In the present study, 3 of the 14 patients admitted before 2010 underwent a partial nephrectomy, whereas 29 of 53 cases admitted after 2010 received a partial nephrectomy.

Intraoperative pathological examination may facilitate accurate diagnosis and help clinicians to modify their surgical approach. However, a minority of CRCC cases may not show any malignant signs on intraoperative pathology. In our study, three CRCC cases were intraoperatively diagnosed as simple renal cysts; these patients required a second anesthesia for radical nephrectomy. Indeed, even with frozen section evaluation at the time of surgery, confusion may remain in the determination of benign versus malignant disease. The cause of inexplicit pathology on frozen biopsy and aspiration is probably due to compression or ischemia of the cyst wall. If there is any intra-operative doubt regarding the absence of neoplasm, the renal cystic mass should be removed with clear margins. If suitable, a renal sparing approach should be considered. Currently, we are attempting to implement this philosophy to all patients.

In our series, 217 of the 2679 RCC patients had concurrent cystic renal lesions, and 4 of the 67 CRCC patients had other concurrent RCCs. The kidneys are prone to a variety of cystic disorders that include developmental, acquired, and neoplastic lesions. However, the synchronous occurrence of two different tumors within the same kidney is a rare event. With the widespread use of ultrasonography, CT, and MRI, such coexisting tumors are now more readily diagnosed. Two or three concurrent renal cell tumors have been reported in cases involving hybrid tumors [14] and Birt-Hogg-Dubé syndrome, but also in sporadic other cases [15]. A recent report by Tyritzis et al. described the combination of two dissimilar RCC subtypes: a chromophobe lesion in the upper pole and a clear cell lesion in the lower pole of the same kidney [16]. The etiology and pathogenesis of such multiple tumors remain unclear. It has been hypothesized that concurrent tumors can arise from tissues with similar embryological origin when they are simultaneously affected by factors such as carcinogens or hormones. The authors assumed that different renal tumors could arise from cancer stem cells that follow dissimilar differentiation pathways regulated by tissue microenvironmental interactions [17]. Other hypotheses are the evolution from one subtype to another (for example, oncocytomas possess the ability to evolve into papillary carcinomas [18]) or the transformation of one malignant renal tumor to another type. The presence of multifocal renal lesions complicates their surgical management. In our opinion, any secondary lesion identified within a kidney should be thoroughly evaluated to minimize the chance of leaving a malignancy behind following a nephron-sparing procedure. If we would choose a partial nephrectomy to treat a solid renal lesion, we should also choose a partial nephrectomy, not an unroofing procedure, to treat non-simple renal cysts, which might avoid a second surgery.

Murad et al. suggested Fuhrman nuclear grade 1 as a defining criterion for CRCC [4]. However, we — along with other investigators — do not favor including grade as a diagnostic characteristic for these tumors [19]. In our study, although most tumors were Fuhrman grade 1, we encountered some CRCC tumors of nuclear grades I-II and II as well.

Cystic renal cell carcinoma carries a better prognosis than other RCCs because of its low nuclear grade and TNM stage regardless of tumor size [20]. The 10-year survival rate and non-recurrence rate after surgery have been reported as 97.3% and 90.3%, respectively [21]. To date, the overall survival in our patient population is more than 90%; with 6–84 months of follow-up, there have not been any signs of local or distant recurrence. Patients initially misdiagnosed as having a benign tumor on pre- or intra-operative pathology that are subsequently diagnosed with a malignant tumor postoperatively should undergo remedial measures (radical nephrectomy) as soon as possible; this situation occurred in 7 cases from our series. Intraoperative cyst rupture during resection did not have any clinical impact on patients’ outcomes after radical nephrectomy; these patients’ outcomes were excellent, with no evidence of neoplastic recurrence or metastasis. Because of the rarity of this disease entity and the limited number of patients reported in the literature and in our study, additional research is needed to further show the diagnostic, pathologic, and prognostic characteristics of CRCC.

## Conclusions

Our results indicate that CRCC is uncommon. CRCC carries an excellent prognosis following surgical treatment. Partial nephrectomy should be regarded as the preferred surgical technique in the management of CRCC.

### Consent

Written informed consent was obtained from the 4 patients (4 cases of concurrent RCCs) for publication of this manuscript and accompanying images. A copy of the written consent is available for review by the Editor-in-Chief of this journal.

## Abbreviations

CRCC: Cystic renal cell carcinoma
RCC: Renal cell carcinoma.
